# AI image analysis as the basis for risk-stratified screening

**DOI:** 10.1007/s11604-025-01734-4

**Published:** 2025-01-11

**Authors:** Fredrik Strand

**Affiliations:** 1 Department of Oncology-Pathology, Karolinska Institutet, Solna, Sweden; 2 Medical Diagnostics Karolinska, Karolinska University Hospital, Solna, Sweden

**Keywords:** Artificial intelligence, Breast cancer screening, Mammography, Risk prediction

## Abstract

Artificial intelligence (AI) has emerged as a transformative tool in breast cancer screening, with two distinct applications: computer-aided cancer detection (CAD) and risk prediction. While AI CAD systems are slowly finding its way into clinical practice to assist radiologists or make independent reads, this review focuses on AI risk models, which aim to predict a patient’s likelihood of being diagnosed with breast cancer within a few years after negative screening. Unlike AI CAD systems, AI risk models are mainly explored in research settings without widespread clinical adoption. This review synthesizes advances in AI-driven risk prediction models, from traditional imaging biomarkers to cutting-edge deep learning methodologies and multimodal approaches. Contributions by leading researchers are explored with critical appraisal of their methods and findings. Ethical, practical, and clinical challenges in implementing AI models are also discussed, with an emphasis on real-world applications. This review concludes by proposing future directions to optimize the adoption of AI tools in breast cancer screening and improve equity and outcomes for diverse populations.

## Introduction

Breast cancer remains a leading cause of cancer-related mortality worldwide, underscoring the critical need for effective early detection strategies [1]. Mammographic screening has long been the cornerstone of early detection, significantly reducing mortality by identifying cancers at a stage amenable to treatment [2, 3]. However, traditional screening programs often adopt uniform protocols based on age, disregarding other risk factors influencing individual risk. This one-size-fits-all approach can lead to significant drawbacks, including overdiagnosis, unnecessary interventions, and, paradoxically, missed cancers in higher-risk individuals. This issue is particularly evident in Japan, where breast cancer incidence peaks in women in their 40s who predominantly have dense breast tissue, leading to the consideration of several supplemental imaging approaches [4].

**Fig. 1:**
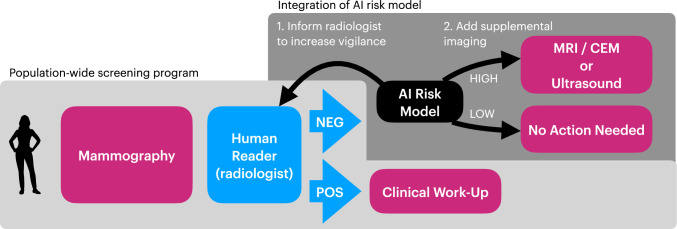
The integration of an AI risk model to inform the human reader of the standard screening modality (mammography) and to identify women for supplemental imaging by Magnetic Resonance Imaging (MRI) or Contrast-Enhanced Mammography (CEM) or Ultrasound. If the human reader does not detect any image signs of potential cancer, i.e., the mammogram is negative, then the mammograms are further analyzed by an AI Risk model. If the model assigns a high risk of cancer, there are several options how to proceed: (i) the human reader may be informed and re-read the mammogram, (ii) the individual may be recalled for contrast-enhaned MRI, contrast-enhanced mammography, or ultrasound. If the AI Risk model assigns a low risk of cancer, no further action would be needed.

Risk-stratified screening offers the possibility of a more nuanced alternative by tailoring supplemental modalities or screening intervals to an individual’s risk profile (Fig. 1). Artificial intelligence (AI) has revolutionized this approach, with two primary applications in breast cancer screening: cancer detection and risk prediction. AI Computer-Aided Detection (CAD) systems analyze mammograms to detect potential malignancies and have demonstrated marked improvements in clinical trials such as ScreenTrustCAD, employing AI as an independent reader, and MASAI (Mammography Screening with Artificial Intelligence trial), employing AI to triage examinations to one or two radiologists [5, 6]. In most countries, several AI CAD systems with regulatory approval are available. In contrast to detecting image signs of potential cancer, AI risk models predict a patient’s likelihood of developing breast cancer over a defined time horizon. Advances in AI, from models leveraging traditional mammographic density to deep learning systems and multimodal frameworks, have significantly enhanced risk prediction accuracy. This review explores the evolution of AI-driven approaches in breast cancer screening, examining their potential for real-world application while addressing key limitations and challenges.

## From imaging biomarkers to deep learning models

Traditional breast cancer risk prediction models relied on mainly questionnaire-type information from patients, and have only recently included mammographic density as an additional risk factor [7]. Interestingly, this type of risk models has largely not required regulatory approval in contrast to the newer models based on AI. Density as a risk factor for breast cancer has been verified in the Japanese population [8].

A first step toward adding image-based risk factors were the studies by Eriksson et al. in the KARMA (The Karolinska Mammography Project for Risk Prediction of Breast Cancer) cohort, showing that adding biomarkers, corresponding to masses and microcalcifications, produced by an AI CAD model increased the accuracy of risk prediction models [9]. The area under the receiver operating characteristics curve (AUC), based on 3-year follow-up time, was 0.63 (95% CI 0.60–0.65) for a regression model with mammographic density as the predictor [8] adjusted for body mass index (BMI) and age at mammography, which increased to 0.64 (95% CI 0.62–0.67) when adding family history of breast cancer and hormone replacement therapy use, and showed a larger increase to 0.71 (95% CI 0.69–0.73) after adding image biomarkers related to microcalcifications and masses. However, the approach was potentially limited by its use of features defined for the purpose of detection, leading to a potential inability to fully capture the risk-relevant complexities of breast tissue architecture.

End-to-end deep learning has transformed breast cancer risk prediction by enabling models to learn directly from the pixel data in mammograms without requiring explicit feature definitions. Unlike AI CAD systems, which focus solely on identifying current abnormalities indicative of malignancy, AI risk models predict the likelihood of future cancer diagnosis, often leveraging subtle imaging patterns correlated with risk factors which may include subtle signs of existing malignancy. In 2019, Yala et al. introduced a convolutional neural network (CNN) trained on more than 70,000 mammograms of which 2732 were from women diagnosed with breast cancer [10]. Compared to the traditional risk model, Tyrer-Cuzick, with an AUC, based on 5-year follow-up time, of 0.62 (95%CI: 0.57–0.66), the image-only deep learning model was considerably more accurate with an AUC of 0.68 (95%CI: 0.64–0.73) which increased to 0.70 (95%CI: 0.66–0.75) after adding traditional risk factors. In addition, they demonstrated how the Tyrer-Cuzick model did not work well for African Americans, AUC 0.45 (95%CI: 0.21–0.66) while the image-only deep learning model consistently achieved an AUC of 0.69 (95%CI: 0.55–0.92). During the following years, the model by Yala et al. was improved, internationally validated and named 'MIRAI' [11–13].

Our research group showed that a CNN-based risk model trained on nearly 10,000 mammograms of which 891 were from women diagnosed with breast cancer outperformed traditional density-based risk models [14]. This study employed a temporal split of the training and test data, aiming to mimic a real-world implementation, implying that the accuracy in the test data would also take into account potential data drift over time, which a random split does not. The AUC, based on 4-year follow-up time, for the deep learning model was 0.65 (95%CI:0.63–0.66) compared to 0.60 (95%CI:0.58–0.61) for an age-adjusted mammographic density model.

In 2019, the risk models from our group and from Yala et al. were based on a single training strategy to predict any future breast cancer based on the input of all images for all individuals. Later, we showed that decoupling the training strategies for cancer detection and the underlying risk enabled a higher accuracy for short-term and medium-term risk prediction compared to a convoluted approach [15]. For the short-term 'cancer signs' model, negative cases were represented by all images from cancer-free individuals and positive cases were represented by ipsilateral images from individuals diagnosed with breast cancer within one year of the image acquisition date. For the medium-term 'underlying risk' model, negative cases were represented by all images from cancer-free individuals and positive cases were represented by contralateral images from any time before diagnosis and ipsilateral images no later than one year before diagnosis.

In 2024, the latest iteration of the model from our group was named 'AISmartDensity' and was based on separate scores, and training strategies, for the three components of underlying risk, masking potential and cancer signs, lending some explainability to the model. Compared to the previous mode, we had added a 'masking' component. The training strategy for the 'masking' component was based on mimicking the subjective visual assessment by five radiologists who reviewed over 10,000 mammograms in total, allocating each one by 'potential to hide cancer' into eight categories [16]. In retrospective evaluation, it showed AUC of 0.67, based on 3-year follow-up time. Along with the models by Yala et al., this represented a significant step forward in leveraging AI as a tool to refine breast cancer screening protocols.

In a paper by Hall et al., the value of integrating polygenic risk scores with mammographic density and lifestyle factors was explored to offer a more holistic approach to personalized risk assessment [17]. Their paper showed the image biomarkers of risk are the most important (AUC 0.73), and the marginal value of adding lifestyle factors (AUC 0.74) and further adding polygenic risk scores (AUC 0.77) was relatively modest.

The collected research on AI risk models underscores the potential of deep learning to extract risk-relevant information from screening mammograms and the potential to further increase the performance by combining genetic predisposition, lifestyle choices, and imaging characteristics.

## Multi-AI retrospective study

Retrospective evaluations are a critical step in assessing the potential real-world performance of AI models. In addition to the single-model retrospective studies reported in the sections above, Arasu et al. conducted a large-scale retrospective study comparing multiple AI models for risk prediction[18]. The findings showed that AI systems consistently outperformed traditional risk models, including density-based methods, in distinguishing high- and low-risk individuals. In this evaluation, the MIRAI model was the only one trained end-to-end for risk prediction based on deep learning modeling of mammograms. MIRAI outperformed the other models with an AUC, based on a 5-year follow-up time, of 0.67 compared to AUC 0.61 for Tyrer-Cuzick, AUC 0.65 for the ProFound risk model based on the work by Eriksson et al. described above, and AUC between 0.63 and 0.65 for three AI CAD models trained for cancer detection.

Acknowledging that AUC is an imperfect metric and hard to interpret in terms of clinical utility, Arasu et al. included a supplemental table describing how many individuals with a 3% or higher risk that models were able to identify, reporting 15% of the population for the MIRAI model albeit at a rather poor calibration with an observed-to-expected ratio of 0.50 compared to the lower 2.6% of the population for the BCSC (Breast Cancer Surveillance Consortium) model (which includes density) at a better calibration with a 1.08 observed-to-expected ratio.

## Prospective clinical trials

Prospective validation is essential for translating any risk model into routine clinical workflows. Several prospective studies have been carried out, demonstrating the ability of supplemental imaging to increase cancer detection. In the Dutch DENSE (Dense Tissue and Early Breast Neoplasm Screening) trial, women with a negative screening mammography, 50 to 75 years of age, and who had BIRADS (Breast Imaging Reporting and Data System) D mammographic density, were invited to the randomized clinical trial [19]. The proportion of interval cancers were more than 80% lower for women undergoing supplemental magnetic resonance imaging (MRI) compared to those who did not undergo MRI. In the Japanese J-START (Japan Strategic Anti-Cancer Randomized Trial), asymptomatic women, 40–49 years of age, were randomized to either mammography and ultrasound or mammography alone [20]. The sensitivity was 91% in the former, and 77% in the latter group. The proportion of interval cancers were reduced by around 50%. The advantages with supplemental imaging are well established. However, the disadvantages include an increased cost for the healthcare system and a burden on the screening participants in terms of requiring further screening visits and the false positive findings. It is critical that the selection method, the risk model, maximizes the additional cancer detection in a reasonably small proportion of the population at highest risk.

**Fig. 2:**
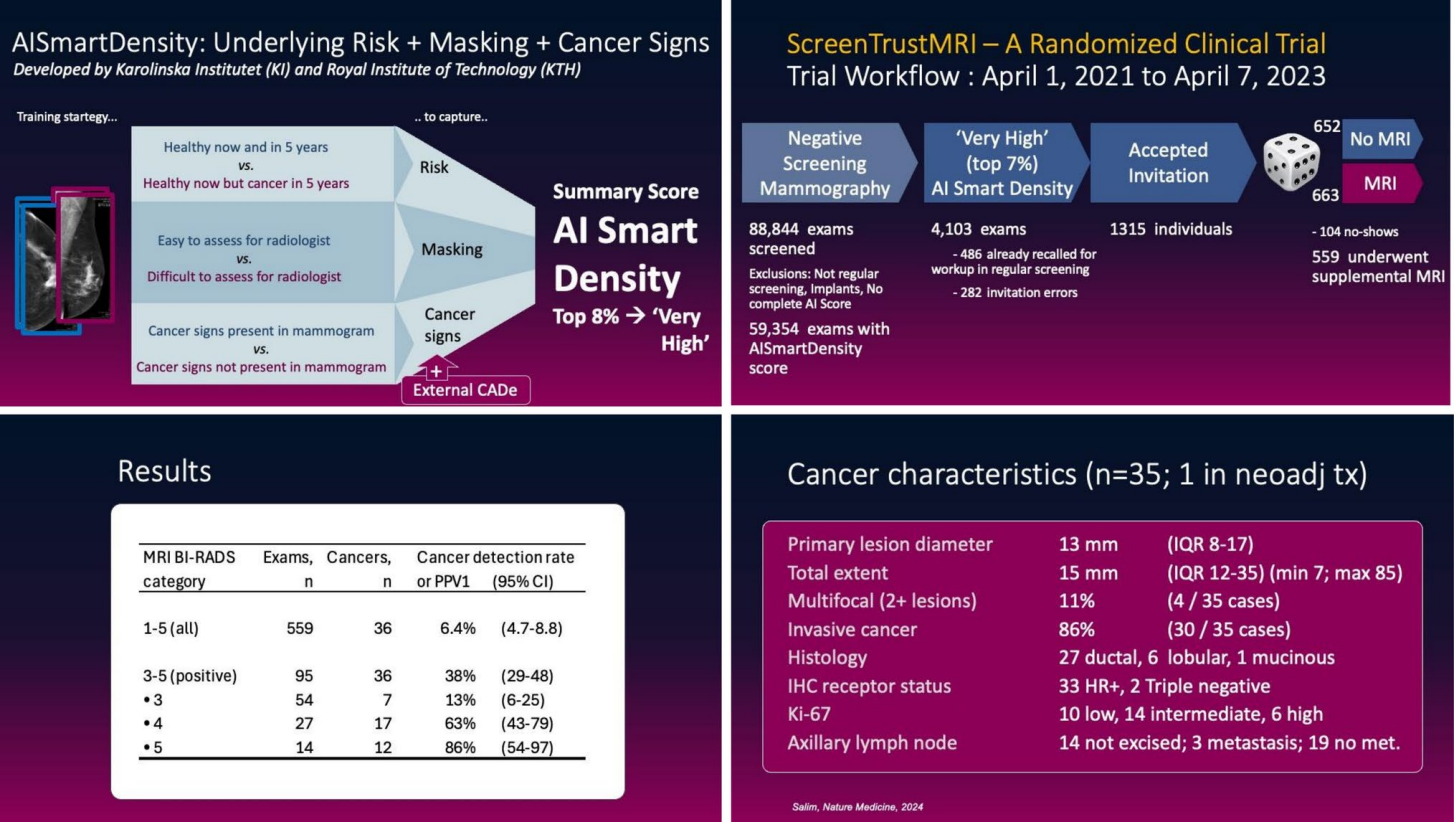
The AI risk model, AISmartDensity, was developed in our group and consists of three components assessing different aspects of the screening mammograms—underlying risk of breast cancer, potential for masking of cancer by normal tissue, and cancer signs. 1315 individuals with negative mammography were included in the randomized clinical trial ScreenTrustMRI.559 underwent MRI. 36 cancers were detected, 86% of which were invasive, with a median size of 13 mm.

While AI CAD systems are increasingly used in screening programs and have achieved regulatory approval in many countries, AI risk models remain mostly in a research setting. However, the randomized ScreenTrustMRI trial from our group exemplifies how AI risk models could be validated in real-world settings by evaluating their effectiveness in triaging patients for supplemental MRI screening [21] (Fig. 2).

**Fig. 3:**
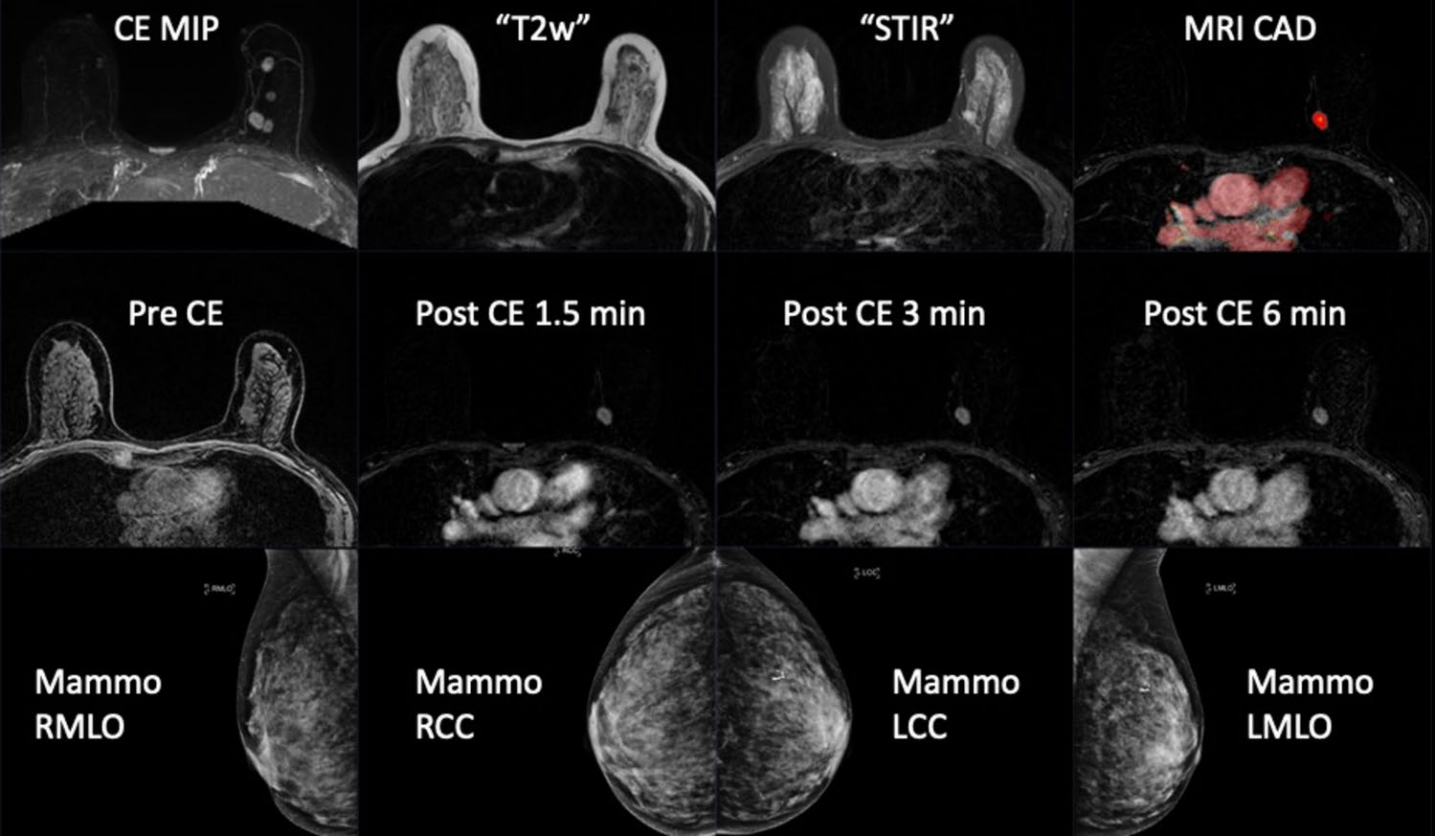
An example of the viewing protocol used for the MRI examinations in the ScreenTrustMRI trial (top and middle row), and the corresponding prior negative screening mammogram (bottom row). The patient was a 70-year-old woman with MRI findings of several contrast-enhancing lesions in her left breast, where the largest one was circumscribed oval, with low-fat signal, intermediate T2 signal, and with heterogeneous wash-out dynamics, which at histopathological analysis corresponded to multifocal invasive ductal cancer nuclear grade 2, ER-positive, HER2-negative, with the largest invasive lesion, 12 mm, shown in the images and a total extent of 68 mm

Based on the AISmartDensity risk model, we invited individuals with negative screening mammograms having the highest AI scores (top 6.9% in a preceding retrospective calibration). In total, 1315 individuals accepted the invitation of which 663 were randomized to have MRI and 652 to not have MRI. In the MRI-group, 559 individuals completed the breast MRI examination, leading to the additional detection of breast cancer among 36 of them. The resulting additional cancer detection rate of 64 cancers per 1000 MRI examinations was more than four times higher than the 16.5 cancers per 1000 MRI examinations in the landmark DENSE trial [22]. The invited proportion of the total population was similar in the DENSE trial as in ScreenTrustMRI. Women were invited if they had 'extremely dense' breasts based on the percentage of mammographic density. The ScreenTrustMRI trial’s results highlight the important gains in cost-per-cancer-detected using AI risk models to suggest supplemental imaging. Figure 3 shows one example of the MRI images that were available to the radiologists. The primary goal of a risk-based cancer screening strategy should be to improve prognosis. Therefore, it is important to review the cancer characteristics of the 36 cancers detected in the trial. The median diameter of the primary invasive lesions was 13 mm, and the range of total extent was between 7 and 85 mm. 86% of the cancers were invasive and only 14% were ductal cancer in situ (DCIS). Four of 35 were multifocal cancers. Three individuals had metastasis to lymph nodes. These characteristics suggest that it was beneficial to detect the cancers now rather than months or years later. However, we will have more information to assess the impact later once we have compared the MRI-group with the no-MRI-group after the 27-month follow-up time.

## Strengths of AI risk models

AI models have achieved remarkable progress in breast cancer risk prediction setting new benchmarks for accuracy and efficiency. These models excel at identifying subtle imaging features and integrating diverse data streams, making them highly adaptable to varied clinical contexts. Additionally, multimodal frameworks combining imaging, genetic, and clinical data add some improvement in risk stratification.

## Limitations and areas for improvement of AI risk models

Despite these strengths, AI models face significant challenges. Many are trained on images from a limited geographical, area , raising some concerns about their applicability to underrepresented populations. In addition, the source of the input data, the mammography equipment may significantly bias the AI model. AI models must be separately validated on various populations and each specific mammography equipment from which images will be analyzed. Additionally, prospective trials like ScreenTrustMRI, though promising, were conducted in resource-rich environments, limiting their direct generalizability to lower-resource settings. A reasonable suggestion might be to replace MRI with contrast-enhanced mammography which would still confer a marked improvement compared to non-contrast enhanced mammography or tomosynthesis [23, 24].

The reliance on black-box algorithms also raises concerns about physician and patient trust, a key barrier to clinical adoption. Efforts to enhance explainability, such as the three-component AISmartDensity from our group, might be important for building clinician and patient trust.

## Tailoring approaches for women with high breast density

The integration of AI risk models into clinical practice offers a unique opportunity to address the challenges associated with high breast density, a well-established risk factor for breast cancer. In the United States, the FDA mandates that women with dense breast tissue receive notification letters, informing them of their density status and potential increased risk of breast cancer. Approximately 40–50% of women undergoing mammography fall into this category [25], creating a significant logistical and clinical challenge for healthcare systems that lack the capacity to offer supplemental screening to such a large population. As insurers gather data on the outcomes of interventions for women with dense breasts, they may determine that the cost is too high and the clinical benefit is too low to justify continued reimbursement for supplemental imaging [26]. This could lead to a backlash against density-based screening policies, undermining the original intent of improving early detection.

In a Japanese context, there was a recent paper by Dr. Uematsu, raising awareness about the seemingly lower effectiveness of screening mammography in Japan compared to e.g., the United States, Europe and Australia. Two important underlying differences were identified. First, the peak of breast cancer incidence is in the 40–49 years age group in Japan compared to 60–69 years in Western settings [4]. Second, in this age group, 90% of Japanese women have heterogeneous or extremely dense breasts compared to 38% of Australian women [27]. The results in the previously discussed J-START trial indicate that adding ultrasound for the 40–49 age group is beneficial. It may be interesting to examine whether an AI risk model could further refine the selection of which women receive ultrasound to include some women with an elevated risk after the age of 49, and possibly to add MRI for higher-risk women in the 40–49 age group.

Instead of relying solely on breast density as a broad categorization, AI systems like AISmartDensity could refine the selection process by being directly trained to predict the outcome of future breast cancer. This precision targeting would allow healthcare facilities to allocate resources more effectively, reducing unnecessary imaging while ensuring that women at the greatest risk receive appropriate intervention.

## Broader integration considerations

The use of AI risk models to tailor supplemental screening for women with dense breasts aligns with broader efforts to integrate AI into clinical workflows. Practical barriers remain, including training radiologists to interpret AI outputs and ensuring interoperability between AI systems and existing radiology infrastructure. Furthermore, healthcare systems must invest in computational resources and user-friendly integration into PACS systems and image viewers.

Efforts to integrate AI-driven risk models should recognize that these tools face distinct barriers compared to AI CAD systems, which are already integrated into a few screening workflows. Compatibility with existing protocols, such as density notifications, will be essential to ensure that AI risk models can effectively complement current screening systems and help reduce the overall burden on the healthcare system. The implementation approach needs to strike a balance between legislative mandates, clinical resources, and patient outcomes, ensuring that breast cancer screening becomes more personalized and effective.

## Future directions

- **Ensuring Generalizability**: Developing diverse, representative datasets and conducting validation studies across varied populations are critical for ensuring model accuracy and calibration.
- **Expanding Multimodal Integration**: Combining imaging with genomic, lifestyle, and clinical data may offer the highest risk prediction accuracy.
- **Addressing Ethical Concerns**: Establishing regulatory frameworks, and well-characterized datasets, to mitigate bias, ensure accountability, and protect patient data is essential for ethical AI deployment.
- **Streamlining Clinical Workflows**: Developing intuitive AI interfaces and providing training for radiologists would facilitate integration into practice.

## Conclusion

AI-driven analysis has shown potential to revolutionize breast cancer screening through two distinct applications: cancer detection (CAD) and risk prediction. While AI CAD systems are increasingly used in clinical practice to assist radiologists or make independent reads, AI risk models have remained mainly in the research setting. However, as shown in retrospective and prospective trials, they offer a potential to complement AI CAD by personalizing screening intervals and supplemental imaging. However, challenges related to generalizability, ethical considerations, and workflow integration must be addressed to realize AI’s full potential.
